# All-suture soft anchors versus metal anchors in arthroscopic rotator cuff repair: a multicenter randomized clinical trial

**DOI:** 10.1097/JS9.0000000000004768

**Published:** 2026-02-17

**Authors:** Renhao Yang, Qingsong Zhang, Chengyu Zhuang, Yin Zhang, Luning Sun, Bin Yuan, Yao Huang, Yushun Fang, Shaohua Zhang, Yanan Li, Yaohua He, Weilin Yu, Haiming Wang, Qingxiang Hu, Hengan Ge, Biao Cheng, Lei Wang

**Affiliations:** a Department of Orthopedics, Sports Medicine Center, Shanghai Institute of Traumatology and Orthopedics, Ruijin Hospital, Shanghai Jiao Tong University School of Medicine, Shanghai, China; b Department of Sport Medicine, Wuhan Fourth Hospital, Puai Hospital Affiliated to Tongji Medical College of Huazhong University of Science and Technology, Wuhan, Hubei, China; c Department of Sports Medicine, Shanghai General Hospital, Shanghai Jiao Tong University School of Medicine, Shanghai, China; d Department of Orthopedics, Jiangsu Province Hospital of Chinese Medicine, Affiliated Hospital of Nanjing University of Chinese Medicine, Nanjing, China; e Department of Orthopedics, Shanghai Sixth People’s Hospital Affiliated to Shanghai Jiao Tong University School of Medicine, Shanghai, China; f Department of Orthopedics, Shanghai Tenth People’s Hospital, Tongji University School of Medicine, Shanghai, China; g Department of Sports Medicine, Tongji Hospital of Tongji University, Shanghai, China

**Keywords:** all-suture soft anchor, arthroscopic rotator cuff repair, metal anchor, safety and efficacy outcomes

## Abstract

**Background:**

The study investigates the safety and efficacy of all-suture soft anchors (ASSA) versus metal anchors (MA) in patients undergoing arthroscopic rotator cuff repair (ARCR) through a randomized controlled trial, assessing whether ASSA’s advantages – reduced bone loss and simplified deployment – can achieve non-inferior clinical outcomes compared to conventional MA.

**Materials and methods:**

We conducted a multicenter randomized controlled trial at five Chinese tertiary hospitals (Jan–Oct 2022), with final follow-up in December 2023. Eligible patients had small-to-medium full-thickness rotator cuff tears, Ellman III bursal-side tears, or Lafosse I–IV subscapularis tears. Participants were randomized 1:1 to ARCR with ASSA or MA, followed at 6 weeks, 3, 6, and 12 months. The outcome was included Constant–Murley Score (CMS), American shoulder and elbow surgeons (ASES) score, visual analog scale (VAS) pain, passive range of motion (PROM), and radiographic assessments (postoperative, 6/12 months). All adverse events were recorded. Finally, 87 patients were randomized (ASSA = 43, MA = 44) and 85 received allocated treatment (42 vs 43).

**Results:**

ASSA showed better midterm (6-month) functional recovery (*P* = 0.031) and pain reduction (*P* = 0.005). There was no difference in ASES, VAS, or adverse events (all *P* > 0.05). After 12 months, both groups showed improved CMS (*P* < 0.001), with no between-group difference (*P* = 0.900). No anchor dislocations observed.

**Conclusion:**

ASSA showed comparable safety and efficacy to MA in ARCR after 12 months of follow-up, with superior improvement in early functional recovery and pain alleviation in midterm (6-month) functional recovery and pain relief, supporting its use as an alternative suture anchor for ARCR.

## Introduction

Rotator cuff tears (RCTs) are the most prevalent upper extremity disease, including a spectrum ranging from tendinopathy to full-thickness tears with arthritic changes. RCTs always result in shoulder pain, weakness, and impaired dynamic motion, such as instability in the glenohumeral joint[1]. Currently, arthroscopic rotator cuff repair (ARCR) is widely used to manage RCTs, which aims to reconstruct the torn rotator cuff and restore shoulder function^[2,3]^.

Over many years, conventional suture anchors have been mainly used in ARCR. These anchors are made of rigid materials like stainless steel and titanium alloy, which have been proven to be effective[4]. Other materials, such as polyether ether ketone (PEEK), biodegradable and absorbable materials (polylactic acid and poly-L-lactic acid), have been developed to improve the design of suture anchors as well. However, even if clinical evidence demonstrated that these suture anchors are effective in maintaining the required physiological load, they are still facing some design challenges^[5–9]^. For metallic suture anchors, concerns of articular cartilage damage, migration, and loosening are common in clinical usage[10]. PEEK suture anchors also have been associated with poor bone integration, while absorbable material suture anchors may be related to adverse reactions such as intraosseous cyst formation and synovitis[11].

All-suture soft anchor (ASSA) represents a new generation of suture anchor technology with similar or better biomechanical characteristics and benefits in a smaller size, reduced displacement risk, and enhanced bone preservation compared to other suture anchors^[12,13]^. They have been found to be equivalent to biocomposite and bioabsorbable suture anchors biomechanically^[14,15]^. In terms of the clinical evidence on ASSA, previous reports have primarily focused on acetabular labral or glenoid labral,^[16,17]^ with less emphasis on RCTs. Additionally, evidence from a randomized clinical trial and some meta-analyses revealed that the ASSA fixation was not associated with long-term clinical or functional improvements after 6 or 12 months postoperatively^[12,18,19]^. There is a paucity of high-level clinical evidence to compare and evaluate the ASSA with other suture anchors.

The purpose of our study was to present the clinical results of ASSAs (Juggerknot^®^ Soft Anchors, Zimmer Biomet, Warsaw, IN, USA) and metal anchors (MAs) (Ti Screw and AllThread Titanium Anchors, Warsaw, IN, USA) in patients undergoing ARCR. This study follows the TITAN Guidelines 2025 for transparency in the use of artificial intelligence, with no AI tools employed[20].

## Materials and methods

### Study design

This was a prospective, multicenter, and randomized controlled clinical trial to evaluate the safety and efficacy of ASSA versus MA in patients undergoing ARCR. The trial protocol was approved by the ethics committee of the five participating centers (Supplemental Digital Content Table 1, available at: http://links.lww.com/JS9/G689). All the participants provided written informed consent. This clinical trial followed the Consolidated Standards of Reporting Trials (CONSORT) reporting guidelines[21].

### Eligibility criteria

A total of 87 patients were enrolled by 8 surgeons across 5 class A tertiary hospitals in China from February 2022 to December 2022. All the inclusion and exclusion criteria are detailed in Supplemental Digital Content Table 2, available at: http://links.lww.com/JS9/G689. Eligible participants were randomized to the investigational group (“All-suture soft anchor, ASSA”) or control group (“Metal anchor, MA”) utilizing a central randomization system. Random allocation sequences were computer-generated by an independent statistician using SAS 9.4 software, with a 1:1 allocation ratio. Participant screening and enrollment were conducted by investigators at each center. Group assignments were executed by principal investigators or sub-investigators via the web system prior to intraoperative eligibility confirmation. This study was performed in compliance with the ethical principles of the Declaration of Helsinki. Institutional review board approval was obtained for each investigational site. All patients provided voluntary informed consent before enrollment.

HIGHLIGHTS

- All-suture soft anchors (ASSA) significantly improved at postoperative midterm (6-month) in pain, strength recovery (as the subgroup for Constant–Murley Score [CMS]), and the shoulder forward flexion compared to metal anchors (MA), with no anchor displacements observed, highlighting its potential for rapid functional rehabilitation.
- ASSA achieved non-inferior functional outcomes (CMS, ASES, VAS) and comparable safety to MAs at postoperative 12 months, with no significant differences in safety outcomes or complications.
- Postoperative rotator cuff integrity assessed via the Sugaya classification (Type I–V) revealed a significantly higher proportion of Type III/IV repairs in the ASSA group compared to the MA group at both 6 and 12 months. Furthermore, the bone edema incidence was higher in the ASSA group at 6 months. Despite these morphological differences, there was no difference in the overall re-tear rates and peri-anchor synovial effusion between the two groups, which did not affect the final functional recovery.

### Interventions

All surgeons adhered to a predefined standardized surgical protocol to minimize inter-operator variability, as detailed below: Under general anesthesia, patients were placed either in the beach-chair position or lateral decubitus, according to the surgeon’s preference. A standard posterior-lateral approach was used as an instrumented channel for diagnostic arthroscopy, along with the conventional anterior-lateral approach. Subacromial decompression was conducted in all patients. Intra-articular pathology was addressed, including the evaluation of the subacromial surface of the rotator cuff. Moreover, during bursectomy to expose the subacromial field, surgeons observed the subacromial space, acromion, coracoacromial ligament, and lesions of the rotator cuff. Surgeons also investigated and recorded the condition of RCTs, including configuration, size, and amount of retraction, as well as any other complex lesions such as labial lesions, bone defects, and coracoacromial ligament lesions. Through arthroscopic exploration, surgeons reconfirmed whether patients met the eligibility criteria. All patients underwent arthroscopic RCT repair with concomitant acromioplasty for impingement morphology, confirmed intraoperatively.

This multicenter randomized controlled trial employed a standardized double-row ARCR technique. Surgeons prepared the tendon footprint through mechanical debridement and cortical abrasion. Patients were randomized to medial row fixation using either 2.9 mm all-suture anchors (ASSA, MaxBraid™ Suture) or 5.0 mm titanium alloy MAs (UHMWPE/PEEK sutures), with lateral row fixation using institution-specific devices (Arthrex/Smith & Nephew/DePuy Synthes). Postoperative structured rehabilitation included 6 weeks of sling immobilization with phased exercises: pendulum movements (0–2 weeks), active-assisted motion (2–6 weeks), and functional training (>6 weeks). All rehabilitation procedures were the same for both groups. Outcomes were assessed at 6 weeks, 3, 6, and 12 months via functional scores (Constant-Murley, ASES, VAS), passive range of motion, and MRI evaluations (T1/T2/PD sequences; metal artifact reduction sequencing for MA group). Blinding of outcome assessors was not feasible. PROM assessors required knowledge of patient history during face-to-face visits. Furthermore, due to the distinct radiographic appearance of the investigational device compared to the control device, central radiologists performing the imaging evaluations were inherently unblinded to treatment group assignment during image analysis.

### Assessment after intervention

The primary measurement was CMS at postoperative 12 months[22]. The secondary endpoints were assessed at multiple time points: CMS at postoperative 6 weeks, 3 months, and 6 months; ASES, VAS, and passive ROM at postoperative 6 weeks, 3 months, 6 months, and 12 months. MRI was performed before discharge to observe the fixation of anchors and assess healing condition (classification of the rotator cuff, bone reactions around the anchors, and retear rate) at 6 and 12 months postoperatively. According to Sugaya’s classification, the assessment categorized the healing condition into five distinct types[23]. Our study also reported all adverse events (AEs), surgery-related AEs, and device-related AEs to present the safety performance of the two types of anchors. Preoperative imaging included X-ray, computed tomography (CT), and magnetic resonance imaging (MRI) for comprehensive assessment of bone/joint positions and soft tissue status. Postoperative follow-up included routine X-rays at discharge and an MRI at 6 and 12 months for rotator cuff healing evaluation.

### Sample size and statistical analysis

With a primary endpoint of CMS at 12 months postoperatively, we hypothesized a similar CMS of 76.42 for both anchors,^[24–26]^ with a standard deviation (SD) of 15.16. Using a one-sided test level (α) of 0.025, statistical power (β) of 0.8, and noninferiority margin (δ) of 10.4 as per Kukkonen *et al*.,[27] the total sample size was calculated to be 44 patients per group, assuming an approximate 20% dropout.

The Full Analysis Set (FAS) included all subjects who provided informed consent and underwent surgery with randomized anchors. For subjects who did not complete CMS at postoperative 12 months, the last observation carried forward method was used to estimate the outcome. Secondary endpoints were analyzed based on the actual observed data without imputation. The Per-Protocol Set (PPS) constituted a subset of the study population that strictly adhered to the study eligibility criteria, followed the procedures defined in the protocol, and contributed to the primary endpoint determination, that is, completed the study.

Baseline characteristics were analyzed descriptively, and group comparisons were made using *t*-tests, Wilcoxon tests, and χ^2^ tests. The primary endpoint was analyzed using covariance analysis, adjusting for preoperative CMS, with non-inferiority if the lower 95% confidence interval of CMS difference was not below the margin.

AEs, including anchor loosening, displacement, and retear of the rotator cuff, were documented and analyzed for correlation with procedures and anchors. SAS 9.40 software was used for the statistical analysis. A *P*-value threshold of less than 0.05 was established to define statistical significance.

## Results

### Baseline characteristics

**Figure 1.**
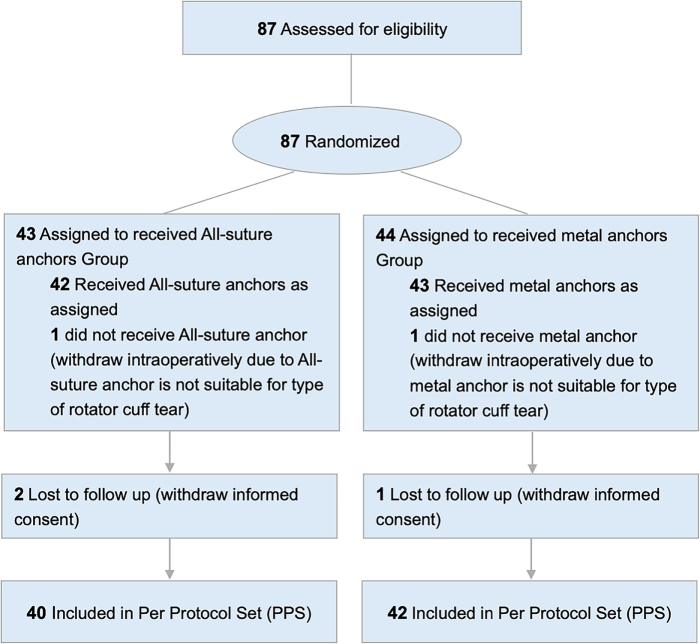
CONSORT flow diagram.

A total of 87 patients were enrolled and randomized into the ASSA group (*n* = 43) or MA group (*n* = 44). Two patients (ASSA group = 1, MA group = 1) were withdrawn intraoperatively because the randomized anchors were not suitable for the type of RCT identified during arthroscopic exploration. Consequently, 85 patients (ASSA group = 42, MA group = 43) underwent arthroscopic repair surgeries using randomized anchors. A total of 82 patients (ASSA group = 40, MA group = 42) have completed the visit schedule per study protocol and were included in the PPS analysis (Fig. 1). Based on FAS analysis, there was an equal distribution of male and female patients (female: 50.00% vs male: 50.00%) in the ASSA group, with a mean (SD) age of 58.52 (9.04) years. MA group comprised a higher proportion of female patients (female: 74.42% vs male: 25.58%) with a mean (SD) age of 60.02 (8.14 years). The analysis revealed no significant differences between the groups in terms of age, height, and BMI distribution (Table 1). Additionally, no statistical differences were found for injury etiology, post-injury management, physical examinations at baseline, and surgical outcomes (Supplemental Digital Content Tables 3 and 4, available at: http://links.lww.com/JS9/G689).

**Table 1:** Baseline characteristics of the trial participants.

Parameter	ASSA group (*n* = 42)	MA group (*n* = 43)	*P* value
Age (years), mean (SD)	58.52 (9.04)	60.02 (8.14)	0.460
Gender, no. (%)			
Male	21 (50.00%)	11 (25.58%)	0.020
Female	21 (50.00%)	32 (74.42%)	
BMI (kg/m^2^), mean (SD)	23.80 (2.78)	24.28 (4.59)	1.000
Injury cause, no. (%)			
Sports injury	6 (14.29%)	3 (6.98%)	0.028
Fall injury	14 (33.33%)	4 (9.30%)	
Traffic injury	1 (2.38%)	3 (6.98%)	
Others	5 (11.90%)	10 (23.26%)	
Unknown	16 (38.10%)	23 (53.49%)	
Post-injury treatment, no. (%)			
Untreated	16 (38.10%)	20 (46.51%)	0.432
Conservative treatment	26 (61.90%)	23 (53.49%)	-
Therapeutic classification			
Drug	18 (69.23%)	16 (69.57%)	0.750
Tricot/bandage brake	0 (0.00%)	1 (4.35%)	
Others	8 (30.77%)	6 (26.09%)	

SD, standard deviation; BMI, body mass index.

### Postoperative CMS scores

**Figure 2.**
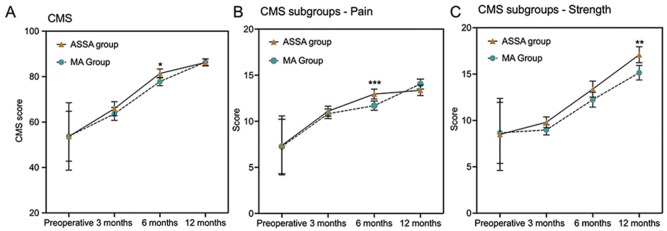
Constant–Murley Score (CMS) results during the study period. (A) Results of the development of the primary outcome, CMS, from baseline to 12 months. (B) Mean CMS profile for the pain group at baseline, 3, 6, and 12 months. (C) Mean CMS profile for the strength group at baseline, 3, 6, and 12 months. Error bars represent the 95% CIs for the means. *P < 0.05, **P < 0.01.

Upon completion of the final follow-up in December 2023, the postoperative CMS was determined from a cohort of 82 patients (ASSA group = 40, MA group = 42). Three patients (ASSA group = 2, MA group = 1) did not complete the postoperative 12-month assessments per protocol and were not included in the PPS for analysis of primary endpoint. No statistically significant differences were found in the total CMS at postoperative 3 and 12 months (*P* > 0.05). At postoperative 6 months, total CMS were 81.42 (1.99) in the ASSA group and 77.85 (1.84) in the MA group. In addition, total CMS score (rate difference: 3.58, 95% CI: 0.34–6.81, *P* = 0.031*), sub score for pain at 6 months (rate difference:1.26, 95% CI: 0.39–2.14, *P* = 0.005***) and sub score for strength at 12 months (rate difference: 3.58, 95% CI: 0.34–6.81, *P* = 0.031*) showed significant differences in the ASSA group compared to the MA group, which showing ASSA group had superior functional recovery and lower pain level results (Fig. 2 and Table 2). The mean (SD) change score of total CMS from baseline was 29.25 (15.27) in the ASSA group and 28.81 (11.11) in the MA group, which showed a statistically significant difference compared to baseline scores in both cohorts (*P* < 0.001) (Table 2).

**Table 2:** Constant–Murley Score (CMS, 0–100) at baseline, postoperative 3, 6, and 12 months.

Outcome mean (SD)	ASSA group (*n* = 40)	MA group (*n* = 42)	Adjusted between-group difference, mean (95% CI)	*P* value
Total CMS at baseline	53.68 (14.84)	53.76 (11.00)	–	0.978
Pain	7.38 (3.20)	7.26 (2.96)	–	0.094
Activities of daily living	10.90 (3.71)	10.71 (3.53)	–	0.759
ROM	24.60 (9.90)	26.10 (7.39)	–	0.439
Strength	8.49 (3.89)	8.66 (3.30)	–	0.844
Total CMS at 3 months	65.87 (3.10)	63.61 (2.85)	2.26 (−2.91, 7.43)	0.386
Pain	11.09 (0.55)	10.82 (0.52)	0.27 (−0.62, 1.17)	0.543
Activities of daily living	14.13 (1.09)	13.44 (1.02)	0.69 (−1.13, 2.51)	0.453
ROM	29.05 (1.95)	28.26 (1.83)	0.79 (−2.51, 4.09)	0.634
Strength	9.81 (0.58)	8.98 (0.54)	0.82 (−0.14, 1.80)	0.094
Total CMS at 6 months	81.42 (1.99)	77.85 (1.84)	3.58 (0.34, 6.81)	0.031^a^
Pain	12.95 (0.54)	11.69 (0.51)	1.26 (0.39, 2.14)	0.005^b^
Activities of daily living	18.05 (1.09)	16.69 (1.02)	1.35 (−0.07, 2.78)	0.063
ROM	35.41 (1.30)	34.84 (1.22)	0.57 (−1.62, 2.77)	0.603
Strength	13.39 (0.86)	12.24 (0.80)	1.15 (−0.25, 2.55)	0.106
Total CMS at 12 months	86.25 (1.55)	86.41 (1.43)	−0.16 (−2.66, 2.35)	0.900
Pain	13.36 (0.57)	14.04 (0.54)	−0.69 (−1.61, 0.24)	0.143
Activities of daily living	17.89 (0.42)	18.54 (0.40)	−0.65 (−1.36, 0.06)	0.070
ROM	36.75 (0.82)	37.46 (0.77)	−0.71 (−2.09, 0.67)	0.309
Strength	17.09 (0.86)	15.15 (0.79)	1.95 (0.57, 3.33)	0.006^b^

CMS, Constant–Murley Score; SD, standard deviation; ROM, range of motion.

^a^ *P* < 0.05.

^b^ *P* < 0.01.

### Postoperative ASES scores

There were no statistically significant differences in the ASES scores at postoperative 3, 6, and 12 months between the groups (*P* > 0.05) (Supplemental Digital Content Table 5, available at: http://links.lww.com/JS9/G689). However, the shoulder forward flexion showed a significant improvement in ASSA group (165.63 ± 22.02) than the degree in MA group (161.25 ± 18.67) at postoperative 6 months (*P* = 0.025).

### MRI evaluation results

The related image is shown in Supplemental Digital Content Figure 1, available at: http://links.lww.com/JS9/G771, and there were no cases of anchor dislocation in both groups based on MRI evaluations until 12 months postoperatively. According to the Sugaya classification[23], the results on repair integrity and retear rates are detailed in Supplemental Digital Content Table 6, available at: http://links.lww.com/JS9/G689. Differences in structural integrity were observed between the ASSA and MA groups at 6 and 12 months postoperatively, with *P*-values of 0.003 and less than 0.001, respectively. Notably, no patients exhibited full-thickness discontinuity (Type V). At 6 months postoperatively, the presentation of peri-anchor effusion was not statistically significant between the groups (*P* = 0.560). At 12 months postoperatively, there were no cases of grades 2 and 3 effusion in the ASSA group, while the MA group had 1 case of grade 3 effusion (*P* = 0.902). Bone edema was observed in 12 (30.00%) cases in the ASSA group at 6 months postoperatively, decreasing to 7 (17.50%) cases at 12 months postoperatively. In the MA group, 2 (5.00%) cases of bone edema were observed, increasing to 6 (14.29%) cases at 12 months postoperatively. Based on logistic regression, adjusted for age (*P* = 0.37) and anchor number (*P* = 0.91), there were no significant predictors of BME (all *P* > 0.05). The difference between the groups was statistically significant at 6 months postoperatively (*P* = 0.006). No case required reoperation during the postoperative follow-up period.

### Safety outcomes

Two anchors in the first patient from the ASSA group pulled out during surgery. The pullout anchors and connected sutures were removed and replaced with new anchors, with no harm to this patient. Investigator analyzed that this may be related to poor bone quality with severe osteoporosis in this patient. Based on the safety set analysis, a total of 26 AEs related to surgery were reported in 15 patients (35.71%) from ASSA group, while 28 AEs were reported in 13 patients (30.23%) from MA group. These 54 AEs were systemic diseases, various reactions, disorders of the connective tissue and musculoskeletal system disorders, including shoulder swelling and pain, joint effusion, constipation, abdominal discomfort, nausea, vomiting, blood oozing, wound secretion, and wound hematoma. There were three serious adverse events in the ASSA group and six in the MA group. Among these, two events (4.65%) in the MA group were assessed to be related to the surgery (one case of rotator cuff syndrome and one case of blood seepage). All events in the ASSA group were considered unrelated to the procedure or anchors.

## Discussion

The results of this non-inferiority and safety clinical study support the clinical performance of ASSA as a safe and effective device in ARCR, addressing it as an effective implant for arthroscopic rotator cuff repair. Moreover, ASSA demonstrated superiority in terms of pain alleviation and muscle strength recovery in the early stages.

MAs are widely used in rotator cuff repair due to their larger size, which can provide enhanced bone hold force after implantation,[28] and have a significant advantage in reducing complications such as inflammation and cyst formation caused by material degradation^[10,29,30]^. Although MA is the oldest anchor type and has slowly been replaced by PEEK, bioabsorbable, or all-suture anchors, it also retains usage due to stable performance and cost-effectiveness in ARCR. However, MA is also associated with potential complications, including anchor displacement, intra-articular embedment of metal implants, bone stress absorption, cartilage damage, and the risk of post-implantation fractures, all of which may lead to revision surgery^[10,28,31]^. Additionally, MA may cause significant artifacts in MRI examinations, thereby affecting the assessment of tendon-to-bone integration and healing after surgery[32]. In our study, we used metallic anchors in 5.0 mm for patients in the control group and found significant improvement in postoperative clinical and functional outcomes. That is comparable to previously published reports^[5,33]^. Although MA is the oldest anchor type and has slowly been replaced by PEEK, bioabsorbable, or all-suture anchors, it also retains usage due to stable performance and cost-effectiveness in ARCR[34].

The considerably smaller soft anchors used for rotator cuff repair offer several advantages. First, since anchor fixation is pointing fixation, more anchors can be used, distributing the tensile loads over multiple points of fixation. Second, this approach simultaneously preserves the surface of the greater tuberosity, enhancing the tendon-to-bone surface area at the time of repair. Our study showed that patients who used ASSAs presented improved mobility since postoperative 3 months compared to those with MAs, particularly in forward flexion and reported lower pain levels. The utilization of ASSA involves the creation of smaller bone tunnels for implantation, potentially leading to reduced compression of the surrounding tissues[35]. This reduction in early postoperative pain can facilitate more effective completions of rehabilitation exercises and consequently result in enhanced mobility during the early recovery phase.

This study observed no anchor displacement in either group, confirming equivalent implantation stability between ASSAs and MAs, consistent with biomechanical studies showing ASSAs achieve sufficient pull-out strength when cortical bone integrity is preserved^[12,18,36,37]^. ASSA performance correlates strongly with cortical bone quality – optimal fixation requires depth adjustment to cortical thickness and perpendicular insertion to avoid oblong tunnel formation^[38,39]^. Biomechanical failures predominantly involve suture rupture rather than anchor pullout if subchondral bone is intact[40], while ASSA’s smaller footprint facilitates revision surgery compared to MAs. Postoperative MRI revealed lower rotator cuff integrity in the ASSA group (3 Type IV retears), however it does not impact the final functional outcome. The elevated incidence of bone edema associated with all-suture anchors might arise from three key mechanisms: localized biomechanical stress concentration inducing trabecular microdamage through cyclic micromotion; enhanced macrophage-mediated inflammatory response triggered by the fibrous nature of ASSA; and potential iatrogenic trauma during tunnel preparation/insertion – whereas rigid metallic anchors demonstrate superior load distribution and osteointegration^[41–43]^.

In summary, ASSA demonstrates comparable or superior mechanical properties performance to MA, with the advantages of a smaller size and lower bone loss. The use of ASSA resulted in lower postoperative pain, promoted early patient rehabilitation, and achieved better clinical outcomes. Findings from our study suggested that ASSA is a viable alternative to MA in ARCR. However, the quality of cortical bone must be taken into consideration, especially in elderly patients with osteoporosis, where further research on the biomechanical performance of ASSA is necessary.

### Limitations

This study has several limitations. First, the enrollment of the first eight patients occurred during the COVID-19 pandemic, necessitating a video-based assessment of the 6-week postoperative PROM. This approach introduced potential inaccuracies, leading to the exclusion of the at this time point. Second, the inability to achieve 90° abduction at 6 weeks precluded the measurement of external rotation in this position, and data in PROMs were not presented. Third, MRI assessments were compromised by artifacts from metallic anchors. Despite systematic comparisons of imaging sequences to evaluate fluid accumulation around the anchors, imaging inaccuracies were unavoidable. Fourth, although 1-year data support the safety and efficacy of all-suture anchors, the elucidation of their characteristic bone tunnel cystic changes’ long-term clinical significance will require an extended follow-up period incorporating MRI, X-ray, and CT imaging at 3 and 5 years to generate more comprehensive and compelling evidence. These additional anchors can complicate the determination of the outcomes of the intended anchors being studied. Future studies should aim to control for these variables to provide more precise assessments of the efficacy of different anchor types.

## Conclusion

In conclusion, ASSA showed comparable clinical and functional results to the MA for ARCR after a follow-up of 12 months. Additionally, ASSA resulted in better shoulder function and pain alleviation at 6 months postoperatively, and superior muscle strength recovery at 12 months postoperatively.

## Data Availability

All datasets used in this study are available from the corresponding author on reasonable request.

## Research registration unique identifying number (UIN)

Chinese Clinical Trial Registry Identifier: ChiCTR2100050143.

## References

[1] Bedi A Bishop J Keener J. Rotator cuff tears. Nat Rev Dis Primers 2024;10:8. 38332156 10.1038/s41572-024-00492-3

[2] Jain NB Ayers GD Koudelkova H. Operative vs nonoperative treatment for atraumatic rotator cuff tears: a trial protocol for the arthroscopic rotator cuff pragmatic randomized clinical trial. JAMA Network Open 2019;2:e199050. 31397866 10.1001/jamanetworkopen.2019.9050 PMC6692688

[3] Abdalla AA. and Pendegrass CJ. Biological approaches to the repair and regeneration of the rotator cuff tendon-bone enthesis: a literature review. Biomater Transl 2023;4:85–103. 38283917 10.12336/biomatertransl.2023.02.004 PMC10817785

[4] Visscher LE Jeffery C Gilmour T. The history of suture anchors in orthopaedic surgery. Clin Biomech (Bristol) 2019;61:70–78. 30502638 10.1016/j.clinbiomech.2018.11.008

[5] Milano G Grasso A Salvatore M. Arthroscopic rotator cuff repair with metal and biodegradable suture anchors: a prospective randomized study. Arthroscopy 2010;26:S112–9. 20692119 10.1016/j.arthro.2010.01.030

[6] Ma R Chow R Choi L Diduch D. Arthroscopic rotator cuff repair: suture anchor properties, modes of failure and technical considerations. Expert Rev Med Devices 2011;8:377–87. 21542709 10.1586/erd.11.4

[7] Tanaka M Hayashida K Kobayashi A Kakiuchi M. Arthroscopic rotator cuff repair with absorbable sutures in the medial-row anchors. Arthroscopy 2015;31:2099–105. 26129724 10.1016/j.arthro.2015.04.094

[8] Byrd JWT. Arthroscopic acetabular labral repair using the Q-FIX suture anchor. Arthrosc Tech 2019;8:e801–e05. 31696043 10.1016/j.eats.2019.03.017 PMC6823793

[9] Suroto H Anindita Satmoko B Prajasari T. Biodegradable vs nonbiodegradable suture anchors for rotator cuff repair: a systematic review and meta-analysis. EFORT Open Rev 2023;8:731–47. 37787481 10.1530/EOR-23-0012 PMC10562948

[10] Kaar TK Schenck RC Jr Wirth MA Rockwood CA Jr. Complications of metallic suture anchors in shoulder surgery: a report of 8 cases. Arthroscopy 2001;17:31–37. 11154364 10.1053/jars.2001.18246

[11] Kramer JD Robinson S Hohn E. Fixation methods and implants in shoulder stabilization: a historical perspective. J Orthop 2018;15:630–35. 29881209 10.1016/j.jor.2018.05.029 PMC5990321

[12] Yang YS Shih CA Fang CJ. Biomechanical comparison of different suture anchors used in rotator cuff repair surgery-all-suture anchors are equivalent to other suture anchors: a systematic review and network meta-analysis. J Exp Orthop 2023;10:45. 37067646 10.1186/s40634-023-00608-w PMC10110812

[13] Du C Chen W Fang J. Comparison of 3 different surgical techniques for rotator cuff repair in a rabbit model: direct suture, inlay suture, and Polyether Ether Ketone (PEEK) suture anchor. Am J Sports Med 2024;52:1428–38. 38619003 10.1177/03635465241240140

[14] Lee J-H Park I Hyun H-S. Comparison of clinical outcomes and computed tomography analysis for tunnel diameter after arthroscopic bankart repair with the all-suture anchor and the biodegradable suture anchor. Arthroscopy 2019;35:1351–58. 30987905 10.1016/j.arthro.2018.12.011

[15] Ro K Pancholi S Son HS Rhee YG. Perianchor cyst formation after arthroscopic rotator cuff repair using all-suture–type, bioabsorbable-type, and PEEK-type anchors. Arthroscopy 2019;35:2284–92. 31350085 10.1016/j.arthro.2019.03.032

[16] Gül O Okutan AE Ayas MS. Arthroscopic glenoid labral lesion repair using all-suture anchor for traumatic anterior shoulder instability: short-term results. J Shoulder Elbow Surg 2019;28:1991–97. 31101476 10.1016/j.jse.2019.03.003

[17] Barber FA Lee Evanson JR. Editorial commentary: acetabular labral repair-is a knotless anchor better? Arthroscopy 2019;35:77–79. 30611369 10.1016/j.arthro.2018.09.003

[18] Yan H Zhao L Wang J. An all-suture anchor offers equivalent clinical performance to an established solid suture anchor in the arthroscopic repair of rotator cuff tears: a prospective, randomized, multicenter trial with 12-month follow-up. Arthroscopy 2024;40:265–76. 37423469 10.1016/j.arthro.2023.06.056

[19] Hoffman TR Lamplot JD McClish SJ. Three medial all suture anchors improves contact force compared to two hard body anchors in a biomechanical two-tendon rotator cuff tear model. Arthrosc Sports Med Rehabil 2022;4:e1601–e07. 36312697 10.1016/j.asmr.2022.05.012 PMC9596862

[20] Agha RA Mathew G Rashid R. Transparency in the reporting of Artificial Intelligence– the TITAN guideline. Prem J Sci 2025;10:100082–85.

[21] Schulz KF Altman DG Moher D Group C. CONSORT 2010 statement: updated guidelines for reporting parallel group randomised trials. Bmj 2010;340:c332. 20332509 10.1136/bmj.c332 PMC2844940

[22] Blonna D Scelsi M Marini E. Can we improve the reliability of the Constant-Murley score? J Shoulder Elbow Surg 2012;21:4–12. 22005124 10.1016/j.jse.2011.07.014

[23] Sugaya H Maeda K Matsuki K Moriishi J. Repair integrity and functional outcome after arthroscopic double-row rotator cuff repair. A prospective outcome study. J Bone Joint Surg Am 2007;89:953–60. 17473131 10.2106/JBJS.F.00512

[24] Lapner P Li A Pollock JW. A multicenter randomized controlled trial comparing single-row with double-row fixation in arthroscopic rotator cuff repair: long-term follow-up. Am J Sports Med 2021;49:3021–29. 34398641 10.1177/03635465211029029 PMC8411465

[25] Kim KC Lee WY Shin HD Han SC. Incomplete articular-side repair increase re-tear rate in full-thickness rotator cuff tears. J Orthop Surg (Hong Kong) 2018;26:2309499018760113. 29486671 10.1177/2309499018760113

[26] Burks RT Crim J Brown N. A prospective randomized clinical trial comparing arthroscopic single- and double-row rotator cuff repair: magnetic resonance imaging and early clinical evaluation. Am J Sports Med 2009;37:674–82. 19204365 10.1177/0363546508328115

[27] Kukkonen J Kauko T Vahlberg T. Investigating minimal clinically important difference for Constant score in patients undergoing rotator cuff surgery. J Shoulder Elbow Surg 2013;22:1650–55. 23850308 10.1016/j.jse.2013.05.002

[28] Longo UG Petrillo S Loppini M. Metallic versus biodegradable suture anchors for rotator cuff repair: a case control study. BMC Musculoskeletal Disorders 2019;20:477. 31653247 10.1186/s12891-019-2834-3 PMC6815043

[29] Jeong JH Shin SJ. Arthroscopic removal of proud metallic suture anchors after Bankart repair. Arch Orthop Trauma Surg 2009;129:1109–15. 19271227 10.1007/s00402-009-0847-3

[30] Silver MD Daigneault JP. Symptomatic interarticular migration of glenoid suture anchors. Arthroscopy 2000;16:102–05. 10627354 10.1016/s0749-8063(00)90136-1

[31] Ozbaydar M Elhassan B Warner JJ. The use of anchors in shoulder surgery: a shift from metallic to bioabsorbable anchors. Arthroscopy 2007;23:1124–26. 17916480 10.1016/j.arthro.2007.05.011

[32] Micic I Kholinne E Kwak JM. Osteolysis is observed around both bioabsorbable and nonabsorbable anchors on serial magnetic resonance images of patients undergoing arthroscopic rotator cuff repair. Acta Orthop Traumatol Turc 2019;53:414–19. 31563430 10.1016/j.aott.2019.08.015 PMC6938998

[33] Papalia R Franceschi F Diaz Balzani L. The arthroscopic treatment of shoulder instability: bioabsorbable and standard metallic anchors produce equivalent clinical results. Arthroscopy 2014;30:1173–83. 24933591 10.1016/j.arthro.2014.03.030

[34] Jain V Gupta H Mehta N. Retrospective comparative analysis of clinical and functional outcome after arthroscopic bankart repair using all-suture anchor and metal anchor. Malays Orthop J 2024;18:11–18. 38638665 10.5704/MOJ.2403.002 PMC11023345

[35] Ntalos D Huber G Sellenschloh K. All-suture anchor pullout results in decreased bone damage and depends on cortical thickness. Knee Surg Sports Traumatol Arthrosc 2021;29:2212–19. 32333058 10.1007/s00167-020-06004-6 PMC8225531

[36] Trofa DP Bixby EC Fleischli JE Saltzman BM. All-suture anchors in orthopaedic surgery: design, rationale, biomechanical data, and clinical outcomes. J Am Acad Orthop Surg 2021;29:e950–e60. 34550098 10.5435/JAAOS-D-20-01224

[37] Minkus M Aigner A Wolke J Scheibel M. All-suture anchor vs. knotless suture anchor for the treatment of anterior shoulder instability-a prospective cohort study. J Clin Med 2024;13:1381. 38592204 10.3390/jcm13051381 PMC10934154

[38] Douglass NP Behn AW Safran MR. Cyclic and load to failure properties of all-suture anchors in synthetic acetabular and glenoid cancellous bone. Arthroscopy 2017;33:977–85e5. 28132809 10.1016/j.arthro.2016.11.022

[39] Ergun S Akgun U Barber FA Karahan M. The clinical and biomechanical performance of all-suture anchors: a systematic review. Arthrosc Sports Med Rehabil 2020;2:e263–e75. 32548592 10.1016/j.asmr.2020.02.007 PMC7283965

[40] Barber FA Herbert MA. All-suture anchors: biomechanical analysis of pullout strength, displacement, and failure mode. Arthroscopy 2017;33:1113–21. 28017468 10.1016/j.arthro.2016.09.031

[41] Tarantino U Greggi C Cariati I. Reviewing bone marrow edema in athletes: a difficult diagnostic and clinical approach. Medicina 2021;57:1143. 34833361 10.3390/medicina57111143 PMC8625152

[42] Kaibara T Kondo E Matsuoka M. Progressive subchondral bone cyst formation following autologous chondrocyte implantation with all-suture anchors: a Case Report with histological evaluation. BMC Musculoskelet Disord 2025;26:115. 39905448 10.1186/s12891-025-08370-z PMC11792339

[43] Uluyardımcı E Öçgüder DA Bozkurt İ. All-suture anchors versus metal suture anchors in the arthroscopic treatment of traumatic anterior shoulder instability: a comparison of mid-term outcomes. Jt Dis Relat Surg 2021;32:101–07. 33463424 10.5606/ehc.2021.75027 PMC8073447

